# SANRA—a scale for the quality assessment of narrative review articles

**DOI:** 10.1186/s41073-019-0064-8

**Published:** 2019-03-26

**Authors:** Christopher Baethge, Sandra Goldbeck-Wood, Stephan Mertens

**Affiliations:** 1 Deutsches Ärzteblatt and Deutsches Ärzteblatt International, Dieselstraße 2, D-50859 Cologne, Germany; 2 Department of Psychiatry and Psychotherapy, University of Cologne Medical School, Cologne, Germany; 3 BMJ Sexual and Reproductive Health, London, UK

**Keywords:** Periodicals as topic, Narrative review articles, Non-systematic review articles, SANRA, Agreement, Reliability, Item-total correlation, Internal consistency, Cronbach’s alpha, Intra-class correlation coefficient

## Abstract

**Background:**

Narrative reviews are the commonest type of articles in the medical literature. However, unlike systematic reviews and randomized controlled trials (RCT) articles, for which formal instruments exist to evaluate quality, there is currently no instrument available to assess the quality of narrative reviews. In response to this gap, we developed SANRA, the Scale for the Assessment of Narrative Review Articles.

**Methods:**

A team of three experienced journal editors modified or deleted items in an earlier SANRA version based on face validity, item-total correlations, and reliability scores from previous tests. We deleted an item which addressed a manuscript’s writing and accessibility due to poor inter-rater reliability. The six items which form the revised scale are rated from 0 (low standard) to 2 (high standard) and cover the following topics: explanation of (1) the importance and (2) the aims of the review, (3) literature search and (4) referencing and presentation of (5) evidence level and (6) relevant endpoint data. For all items, we developed anchor definitions and examples to guide users in filling out the form. The revised scale was tested by the same editors (blinded to each other’s ratings) in a group of 30 consecutive non-systematic review manuscripts submitted to a general medical journal.

**Results:**

Raters confirmed that completing the scale is feasible in everyday editorial work. The mean sum score across all 30 manuscripts was 6.0 out of 12 possible points (SD 2.6, range 1–12). Corrected item-total correlations ranged from 0.33 (item 3) to 0.58 (item 6), and Cronbach’s alpha was 0.68 (internal consistency). The intra-class correlation coefficient (average measure) was 0.77 [95% CI 0.57, 0.88] (inter-rater reliability). Raters often disagreed on items 1 and 4.

**Conclusions:**

SANRA’s feasibility, inter-rater reliability, homogeneity of items, and internal consistency are sufficient for a scale of six items. Further field testing, particularly of validity, is desirable. We recommend rater training based on the “explanations and instructions” document provided with SANRA. In editorial decision-making, SANRA may complement journal-specific evaluation of manuscripts—pertaining to, e.g., audience, originality or difficulty—and may contribute to improving the standard of non-systematic reviews.

## Background

Narrative review articles are common in the medical literature. Bastian et al. found that they constitute the largest share of all text types in medicine and they concluded that they “remain the staple of medical literature” [1]. Narrative reviews also appear popular among both authors and readers, and it is plausible to assume that they exercise an enormous influence among doctors in clinical practice and research. However, because their quality varies widely, they have frequently been compared in blanket, negative terms with systematic reviews.

We use the term narrative review to refer to an attempt to summarize the literature in a way which is not explicitly systematic, where the minimum requirement for the term systematic relates to the method of the literature search, but in a wider sense includes a specific research question and a comprehensive summary of all studies [2].

While systematic reviews are not per se superior articles and while certain systematic reviews have been criticized lately [3], non-systematic reviews or narrative reviews have been widely criticized as unreliable [1, 4]. Hence, the hierarchy of evidence-based medicine places systematic reviews much higher than non-systematic ones. However, it is likely—and even desirable—that good quality narrative reviews will continue to play an important role in medicine: while systematic reviews are superior to narrative reviews in answering specific questions (for example, whether it is advisable to switch an antidepressant among antidepressant non-responders in patients with major depressive disorder [5]), narrative reviews are better suited to addressing a topic in wider ways (for example, outlining the general principles of diagnosing and treating depression [6]).

Critical appraisal tools have been developed for systematic reviews (e.g., AMSTAR 2 [A MeaSurement Tool to Assess Systematic Reviews] [7]) and papers on RCTs (e.g., the CASP [Critical Appraisal Skills Program] checklist for randomized trials [8]) and other types of medical studies. For narrative reviews, in contrast, no critical appraisal, or quality assessment tool is available. Such a tool, however, if simple and brief enough for day-to-day use, may support editors in choosing or improving manuscripts, help reviewers and readers in assessing the quality of a paper, and aid authors in preparing narrative reviews. It may improve the general quality of narrative reviews.

As a consequence, we have developed SANRA, the Scale for the Assessment of Narrative Review Articles, a brief critical appraisal tool for the assessment of non-systematic articles. Here, we present the revised scale and the results of a field test regarding its feasibility, item-total correlation, internal consistency, reliability, and criterion validity.

## Methods

SANRA was developed between 2010 and 2017 by three experienced editors (CB, SGW, and SM) working at a general medical journal, *Deutsches Ärzteblatt*, the journal of the *German Medical Association* and the *National Association of Statutory Health Insurance Physicians*. It is intended to be a simple and brief quality assessment instrument not only to assist editors in their decisions about manuscripts, but also to help reviewers and readers in their assessment of papers and authors in writing narrative reviews.

Two earlier, seven-item versions of SANRA have been developed and tested by the authors, the first in 10 narrative reviews from the field of neurology as retrieved through a PubMed search, the second among 12 consecutive narrative reviews submitted to *Deutsches Ärzteblatt*—both showing satisfactory internal consistency and inter-rater reliability [9].

The current version of SANRA [10] has been revised by the authors in 2014 in order to simplify the scale and make it more robust. We simplified the wording of the items, and we deleted an item addressing a manuscript’s writing and accessibility because ratings of that item differed considerably. The six items that form the revised scale are rated in integers from 0 (low standard) to 2 (high standard), with 1 as an intermediate score. The maximal sum score is 12.

**Fig. 1:**
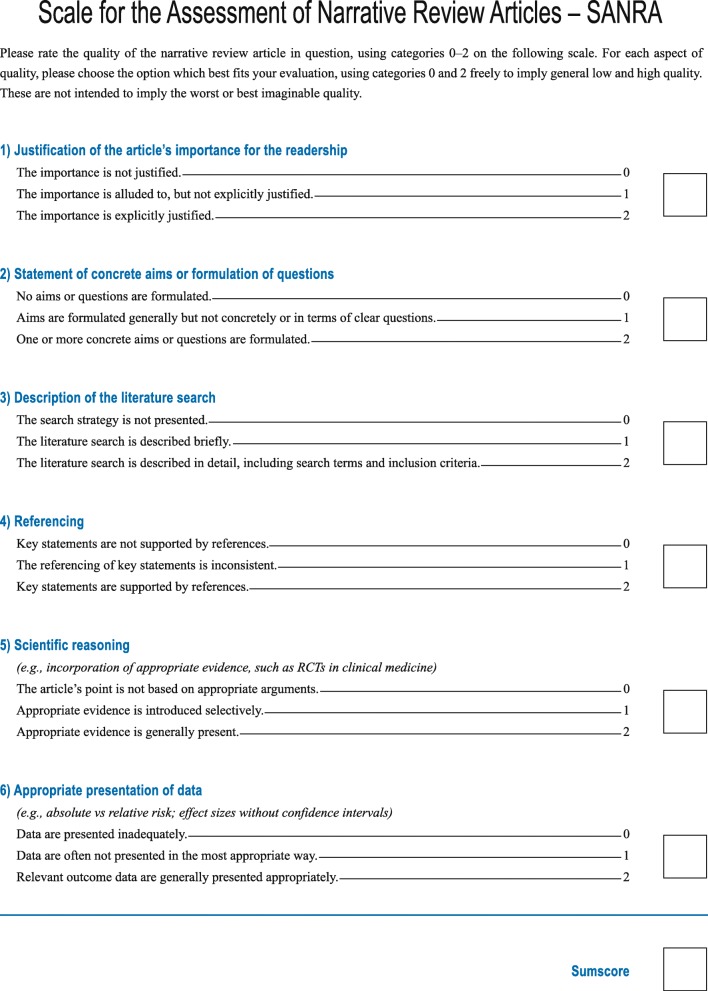
SANRA - Scale

**Fig. 2:**
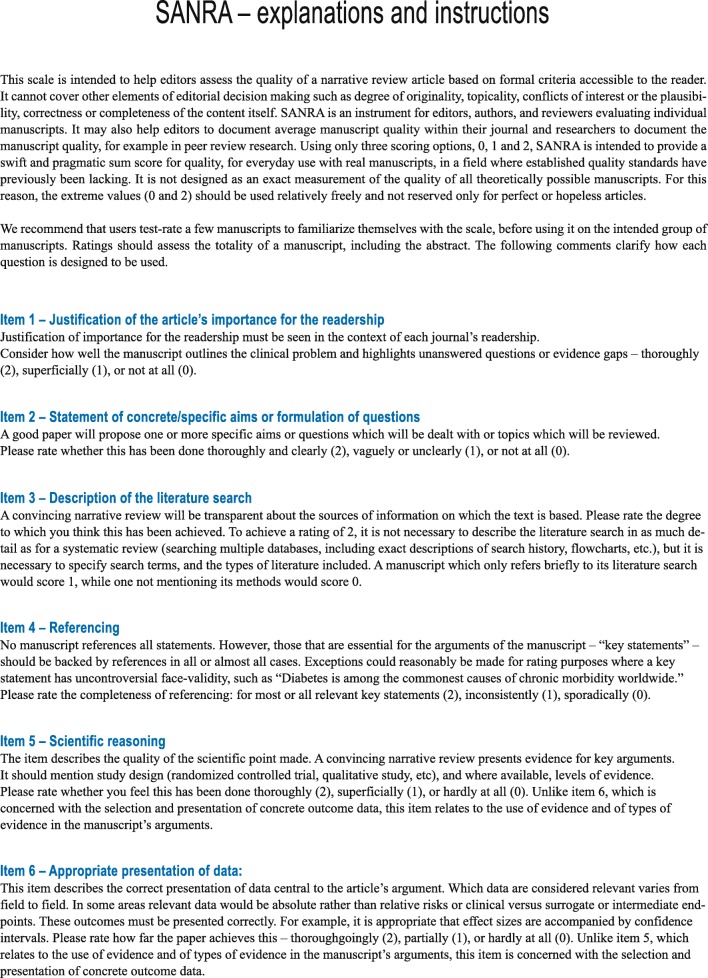
SANRA—explanations and instructions document

The sum score of the scale is intended to measure the construct “quality of a narrative review article” and covers the following topics: explanation of the review’s importance (item 1) and statement of the aims (item 2) of the review, description of the literature search (item 3), referencing (item 4), scientific reasoning (item 5), and presentation of relevant and appropriate endpoint data (item 6) (Fig. 1). For all items, we developed anchor definitions and examples to guide users in filling out the instrument, provided in the document “explanations and instructions,” accompanying the scale. This document was also edited to improve clarity (Fig. 2).

In 2015, one rater (CB) screened all submissions to *Deutsches Ärzteblatt* in 2015, and the first 30 consecutive review manuscripts without systematic literature searches were selected for inclusion in the present study. All three raters (CB, SGW, and SM) are editors, with, in 2015, at least 10 years of experience each. They scored the manuscripts independently and blinded to each other’s ratings.

### Statistical analysis

Descriptive data are shown as means or medians, as appropriate, and as ranges, standard deviations, or confidence intervals. This study aimed at testing SANRA’s internal consistency (Cronbach’s alpha) and the item-total correlation—indicating whether the items measure the same phenomenon, here different aspects of review paper quality—as well as SANRA’s inter-rater reliability with regard to its sum score. Inter-rater reliability, as a measure of the consistency among different raters, was expressed as the average measure intra-class correlation, ICC, using a two-way random effects model (consistency definition). As an approximation of SANRA’s criterion validity (Is the score predictive of other indicators of paper quality, e.g., acceptance and rejection or citations?), we analyzed post hoc whether average sum scores of SANRA were associated with the decision to accept or reject the 30 manuscripts under study (point biserial correlation for the association between a dichotomous and a continuous variable). All calculations were carried out using SPSS. Where possible, the presentation follows the recommendations of the Guidelines for Reporting Reliability and Agreement Studies (GRRAS) [11].

## Results

All 90 ratings (3 raters × 30 manuscripts) were used for statistical analysis. The mean sum score across all 30 manuscripts (*N* = 90) was 6.0 out of 12 possible points (SD 2.6, range 1–12, median 6). Highest scores were rated for item 4 (mean 1.25; SD 0.70), item 2 (mean 1.14; SD 0.84), and item 1 (mean 1.1; SD 0.69) whereas items 6, 5, and 3 had the lowest scores (means of 0.81 (SD 0.65), 0.83 (SD 0.67), and 0.84 (SD 0.60), respectively) (all single-item medians: 1).

The scale’s internal consistency, measured as Cronbach’s alpha, was 0.68. Corrected item-total correlations ranged from 0.33 to 0.58 (Table 1). Tentative deletions of each item to assess the effect of these on consistency showed reduced internal consistency with every deleted item (0.58–0.67) (as shown by the alpha values in Table 1).

**Table 1:** Item-total correlation and impact on Cronbach’s alpha if the items were deleted

Item	Item-total correlation	Alpha if item deleted
Item 1	0.34	0.66
Item 2	0.34	0.67
Item 3	0.33	0.66
Item 4	0.40	0.64
Item 5	0.51	0.60
Item 6	0.58	0.58

Across 180 single-item ratings (6 items × 30 manuscripts), the maximum difference among the 3 raters was 2 in 12.8% (*n* = 23; most often in items 1, 2, and 4), in 56.7% (*n* = 102), the raters differed by no more than 1 point, and in 30.6% (*n* = 55), they entirely agreed (most often in items 2 and 3). The intra-class correlation coefficient (average measure) amounted to 0.77 [95% CI 0.57, 0.88; *F* 4.3; df 29, 58]. Disagreements most often occurred with regard to items 1 and 4.

Average SANRA sum scores of the 30 manuscripts were modestly associated with the editorial decision of acceptance (mean score 6.6, SD 1.9; *n* = 17) or rejection (mean score 5.1, SD 2.1; *n* = 13): point biserial correlation of 0.37 (*t* = 2.09, df 28; two-sided *p* = 0.046).

All raters confirmed that completing the scale is feasible in everyday editorial work.

## Discussion

This study yielded three important findings: (1) SANRA can be applied to manuscripts in everyday editorial work. (2) SANRA’s internal consistency and item-total correlation are sufficient. (3) SANRA’s inter-rater reliability is satisfactory.

### Feasibility

It is our experience with the current and earlier SANRA versions that editors, once accustomed to the scale, can integrate the scale into their everyday routine. It is important, however, to learn how to fill out SANRA. To this end, together with SANRA, we provide definitions and examples in the explanations and instructions document, and we recommend that new users train filling out SANRA using this resource. Editorial teams or teams of scientists and/or clinicians may prefer to learn using SANRA in group sessions.

### Consistency and homogeneity

With Cronbach’s alpha of 0.68 and corrected item-total correlations between 0.33 and 0.58, we consider the scale’s consistency and item homogeneity sufficient for widespread application. It should be noted that because coefficient alpha increases with the number of items [12], simplifying a scale by reducing the number of items—as we did—may decrease internal consistency. However, this needs to be balanced against the practical need for brevity. In fact, the earlier seven-item versions of SANRA had higher values of alpha: 0.80 and 0.84, respectively [9]. Still, the number of items is not necessarily the only explanation for differences in alpha values. For example, the manuscripts included in the two earlier studies may have been easier to rate.

### Inter-rater reliability

The scale’s intra-class correlation (0.77 after 0.76 in [9]) indicates that SANRA can be used reliably by different raters—an important property of a scale that may be applied for manuscript preparation and review, in editorial decision-making, or even in research on narrative reviews. Like internal consistency, reliability increases with the number of items [12], and there is a trade-off between simplicity (e.g., a small number of items) and reliability. While the ICC suggests sufficient reliability, however, the lower confidence limit (0.57) does not preclude a level of reliability normally deemed unacceptable in most applications of critical appraisal tools. This finding underscores the importance of rater training. Raters more often disagreed on items 1 and 4. After the study, we have therefore slightly edited these items, along with items 5 and 6 which we edited for clarity. In the same vein, we revised our explanations and instructions document.

It is important to bear in mind that testing of a scale always relates only to the setting of a given study. Thus, in the strict sense, the results presented here are not a general feature of SANRA but of SANRA filled out by certain raters with regard to a particular sample of manuscripts. However, from our experience, we trust that our setting is similar to that of many journals, and our sample of manuscripts represents an average group of papers. As a consequence, we are confident SANRA can be applied by other editors, reviewers, readers, and authors.

### Validity

In a post hoc analysis, we found a modest, but statistically significant correlation of SANRA sum scores with manuscript acceptance. We interpret this as a sign of criterion validity, but emphasize that this is both a post hoc result and only a weak correlation. The latter, however, points to the fact that, at the level of submitted papers, other aspects than quality alone influence editorial decision-making: for example, whether the topic has been covered in the journal recently or whether editors believe that authors or topics of manuscripts have potential, even with low initial SANRA scores. SANRA will therefore often be used as one, and not the only, decision aid. Also, the decision to accept a paper has been made after the papers had been revised.

Moreover, additional results on criterion validity are needed, as are results on SANRA’s construct validity. On the other hand, SANRA’s content validity, defined as a scale’s ability to completely cover all aspects of a construct, will be restricted because we decided to limit the scale to six items, too few to encompass all facets of review article quality—SANRA is a critical appraisal tool and not a reporting guideline. For example, we deleted an item on the accessibility of the manuscript. Other possible domains that are not part of SANRA are, for example, originality of the manuscript or quality of tables and figures. These features are important, but we believe the six items forming SANRA are a core set that sufficiently indicates the quality of a review manuscript and, at the same time, is short enough to be applied without too much time and effort. SANRA’s brevity is also in contrast to other tools to assess articles, such as AMSTAR 2, for systematic reviews, or, to a lesser extent, CASP for RCTs, with its 16 and 11 items, respectively.

Throughout this paper we have referred to the current version of SANRA as the revision of earlier forms. This is technically true. However, because it is normal that scales go through different versions before publication and because this paper is first widespread publication of SANRA, we propose to call the present version simpy SANRA.

While medicine has achieved a great deal in the formalization and improvement of the presentation of randomized trials and systematic review articles, and also a number of other text types in medicine, much less work have been done with regard to the most frequent form of medical publications, the narrative review. There are exceptions: Gasparyan et al. [13], for example, have provided guidance for writing narrative reviews, and Byrne [14] as well as Pautasso [15] has written, from different angles, thoughtful editorials on improving narrative reviews and presented lists of key features of writing a good review—lists that naturally overlap with SANRA items (e.g., on referencing). These lists, however, are not tested scales and not intended for comparing different manuscripts. SANRA can be used in comparisons of manuscripts the way we used it in our editorial office, that is, in one setting. At the present time, however, it seems unwise to compare manuscripts across different settings because, so far, there are no established cut-offs for different grades of quality (e.g., poor-fair-moderate-good-very good). Still, in our experience, a score of 4 or below indicates very poor quality.

### Limitations

The main limitation of this study is its sample size. While, in our experience, a group of 30 is not unusual in testing scales, it represents a compromise between the aims of representativeness for our journal and adequate power and feasibility; it took us about 6 months to sample 30 consecutive narrative reviews. Also, in this study, the authors of the scale were also the test-raters, and it is possible that inter-rater reliability is lower in groups less familiar with the scale. As for most scales, this underscores the importance of using the instructions that belong to the scale, in the present case the explanations and instructions document. It is also advisable to train using the scale before applying SANRA for manuscript rating. In addition, by design, this is not a study of test-retest reliability, another important feature of a scale. Finally, as previously acknowledged, although we believe in the representativeness of our setting for medical journals, the present results refer to the setting of this study, and consistency and reliability measures are study-specific.

## Conclusion

We present SANRA, a brief scale for the quality assessment of narrative review articles, the most widespread form of article in the medical literature. We suggest SANRA can be integrated into the work of editors, reviewers, and authors. We encourage readers to consider using SANRA as an aid to critically appraising articles, and authors to consider its use on preparing narrative reviews, with a view to improving the quality of submitted and published manuscripts.

SANRA and its explanations and instructions document are available (open access) at: https://www.aerzteblatt.de/down.asp?id=22862, https://www.aerzteblatt.de/down.asp?id=22861.

## Abbreviations

AMSTAR 2: A MeaSurement Tool to Assess Systematic Reviews
CASP: Critical Appraisal Skills Program
GRRAS: Guidelines for Reporting Reliability and Agreement Studies
ICC: Intra-class correlation
RCT: Randomized controlled trial
SANRA: Scale for the Assessment of Narrative Review Articles
